# Comparative analysis of the male inflorescence transcriptome profiles of an *ms22* mutant of maize

**DOI:** 10.1371/journal.pone.0199437

**Published:** 2018-07-13

**Authors:** Yonggang Gao, LiJuan Zhang, ShengChao Zhao, Yuanxin Yan

**Affiliations:** Nanjing Agricultural University, Nanjing, Jiangsu, China; Wuhan University, CHINA

## Abstract

In modern agricultural production, maize is the most successful crop utilizing heterosis. *712C-ms22* is an important male sterile material in maize. In this study, we performed transcriptome sequencing analysis of the V10 stage of male inflorescence. Through this analysis, 27.63 million raw reads were obtained, and trimming of the raw data revealed 26.63 million clean reads, with an average match rate of 94.64%. Using Tophat software, we matched these clean reads to the maize reference genome. The abundance of 39,622 genes was measured, and 35,399 genes remained after filtering out the non-expressed genes across all the samples. These genes were classified into 19 categories by clusters of orthologous groups of protein annotation. Transcriptome sequencing analysis of the male sterile and fertile *712C-ms22* maize revealed some key DEGs that may be related to metabolic pathways. qRT-PCR analysis validated the gene expression patterns identified by RNA-seq. This analysis revealed some of the essential genes responsible for pollen development and for pollen tube elongation. Our findings provide useful markers of male sterility and new insights into the global mechanisms mediating male sterility in maize.

## 1.Introduction

Maize (*Zea mays* L.)is widely grown across the globe and constitutes the world’s largest food crop, exceeding rice, wheat and other cereals in terms of importance since 2012[1]. Maize is consumed in large quantities by both humans and animals, and has a number of industrial applications such asitsuse as a biofuel[2]. As an exemplar plant species, important research is frequently performed on maize, including studies of domestication, molecular evolution and genome architecture[3].

Over 11 million years ago, the maize genome was duplicated through an ancient polyploidy event, and the modern maize genome is replete with chromosomal duplications and repetitive DNA. In modern agricultural production, maize is the most successful crop that utilizes heterosis[4–5].

With advances in technology and with the completion of the maize B73 self-bred line genome sequence, a number of genomic, transcriptomic, epigenomic and proteomic approaches have increasingly been employed to provide new insights into the molecular mechanisms of plants[6]. Among these techniques, transcriptome analysis is a holistic approach that strives to understand the role and the interaction of individual biological components in phenotypic characteristics[7].

In recent years, advances in transcriptome sequencing, or RNA-seq, have allowed for the high throughput analysis of the transcriptome profiles of model plants[8–9], such as *Arabidopsis thaliana*, rice and maize, as well as other important crops such as soybeans, wheat, barley, *Medicago*, *Sorghum* and *Setariaitalica*[10–12]. By using transcriptome analysis in plants, a complete set of transcripts in a cell at a specific developmental stage or under specific physiological conditions can be obtained[13]; this set can then provide information on differentially expressed gene (DEG) levels and gene regulation for a specific pathway of interest[14]. Transcript analysis technology also allows for the direct comparison and the analysis of thousands of genes within one experiment[15]. By comparing DEGs, global transcriptome profiling analysis provides a mechanism with which to identify gene net works for the discovery of functional connections[16].

Yan and colleagues (2012) conducted genome-wide transcriptome sequencing analysis of the young floral buds of the *Brassicanapus* cytoplasmic male sterility (CMS)line Nsa and its novel restorer line NR1 and found a group of candidate genes associated with male sterility[17]. Similarly, Liu and colleagues (2013) employed Solexa/Illumina techniques to analyze the DEGs between the male sterile line 121A and its near isogenic line 121C in peppers. The comparison and analysis of these plant materials found a group of key genes and found significant pathways associated with male sterility[18]. Hao(2016)employed transcriptome profile analysis for drought-tolerant RIL70 and drought-sensitive RIL93 maize recombination inbred lines and detected DEGs between different stages of drought stress in these two lines, providing insights into the dynamic mechanisms underlying drought tolerance in maize seedlings[19–20].

New technological advances are required to increase the yield of hybrid seed production in maize[21]. Therefore, a break through in maize heterosis is required, similar to that which occurred for rice breeding in China. CMS and GMS are common among most plants, and male sterility exists widely among crops[22]. Male sterile lines are useful in the study of plant reproductive development, and these materials can be used as effective tools for hybrid seed production through heterosis. Moreover, GMS plays an important role in the production of hybrid maize seeds[23].

Therefore, the study of male sterility is valuable not only in terms of our theoretical understanding but also in terms of practical applications. However, the molecular mechanism of GMS in maize remains unclear. Although RNA-seq has been employed to examine a variety of plant species, few reports exist on the application of this technology in the study of GMS in maize.

In this study, the *712C*-*ms22*mutantline was selected as the research material to examine the molecular genetic mechanism of GMS in maize. We performed comparative transcriptome analysis of *712C*-*ms22* mutant male inflorescence(V10) using Illumina sequencing technology to identify the DEGs and metabolic pathways that play a role in maize GMS. Our findings advance our understanding of the molecular mechanisms of male sterility in maize and may contribute towards technological advances, for example, in hybrid seed production and in other male sterility lines of maize.

## 2. Results

### 2.1. Characterizing the staminate inflorescences of fertile and sterile *712C*-*ms22* lines

**Fig 1:**
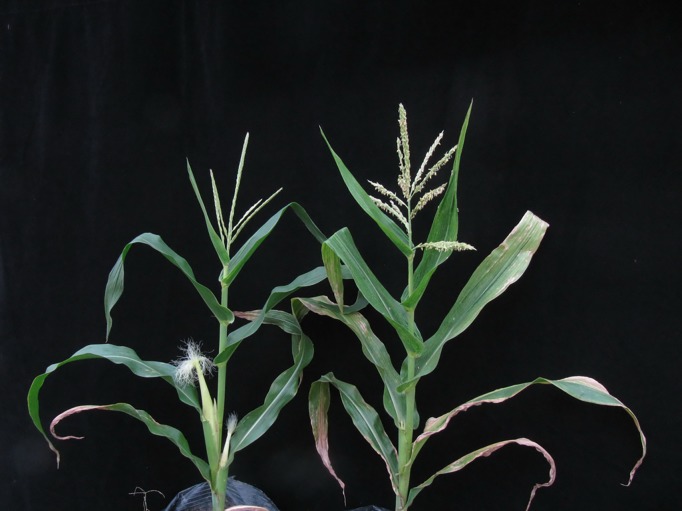
The phenotypes of the sterile and fertile plants of *712C*-*ms22*. a: One of the sterile plants of *712C*-*ms22*; b: One of the fertile plants of *712C*-*ms22*.

Before the reproductive stage, the *712C*-*ms22* mutant plants were normal in appearance, showing no significant differences in phenotype compared to wild-type plants. At a later stage of reproductive growth, the fertile plants showed normal staminate inflorescences, cracked florets and the presence of pollen grains. However, compared with the fertile plant, the staminate inflorescences of sterile *712C*-*ms22* plants were abnormal in appearance, with tightly folded inflorescences and small, thin florets (Fig 1).

The mutant sterile males were smaller and had fewer branches than the normal fertile males.Male sterile (Clever shell)plants possessed tightly closed staminate flowers, whereas fertile (Clever shell) plants possessed loose staminate flowers that are easily cracked.Normal fertile plants were light green to pale yellow in color, whereas sterile plants were light green.

We also found that the ear and filament of female plants grew faster than those of male sterile plants. The ear growth of the fertile plants was slow, and the filaments were shorter. For cultivable plants,the filaments were not drawn out and were observed to be completely in powder form. The ears of fertile plants were short, whereas the fruit ears of sterile plants were longer with denser filaments. This phenomenon may be related to the nutritional distribution of reproductive growth.

**Fig 2:**
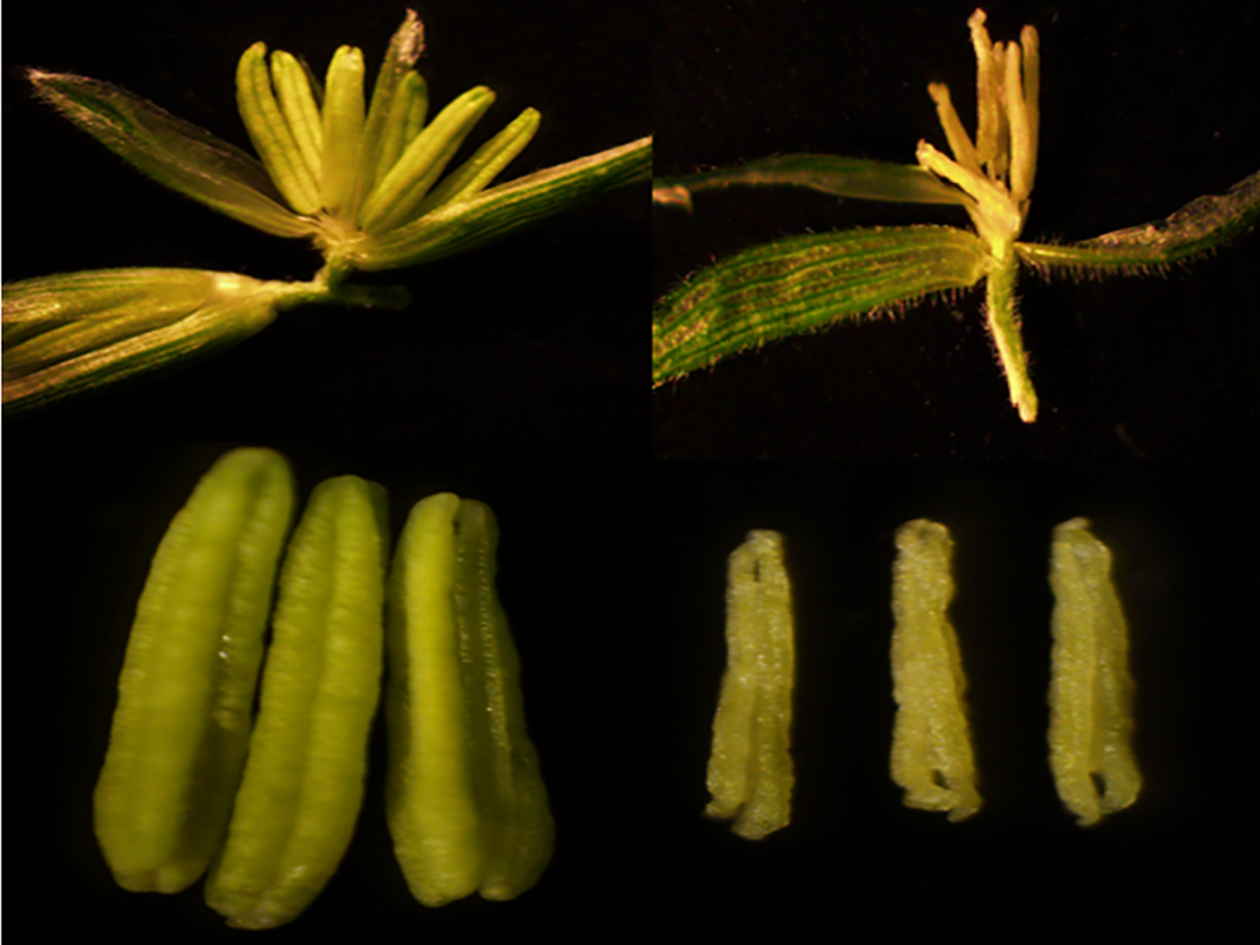
Morphological differences between the male fertile and sterile lines of *712C-ms22*. a: The sterile anther of *712C-ms22*;b: The fertile anther of *712C-ms22*.

After dissecting the fertile and sterile *712C-ms22* plants, phenotypic differences became evident(Fig 2). The fertile plant has normal anthers. In fertile flowers, the anther was plump,uniform, smooth and yellow in color, whereas in the sterile plant, the anther was shriveled, and the flowers were abnormal in appearance.

### 2.2.Transcriptome profile of maize in *712C-ms22*

The cDNA libraries of the staminate inflorescences of *712C*-*ms22* sterile and fertile plants at the V10stage of development were prepared and sequenced using the IlluminaHiSeq 2500 platform. Three biological replicates for each sample were sequenced to increase the statistical power of the analysis. Reads of low sequencing quality were filtered out, and 53.34 million and 42.63 million 100 bp paired-end reads were obtained for the fertile and sterile materials, respectively. Of these reads, 78.59% mapped to unique positions. In total, 79.33% (209 million) of the total paired-end reads were aligned to the B73 reference genome (ZmB73_RefGen_v3). After trimming the raw data, 27.63million raw reads and 26.42million clean reads were obtained for the male inflorescence sample (Table 1). Uniquely mapped reads were used to estimate the transcript levels. Expression values were expressed in units of reads per kilobase per million reads mapped (RPKM). As expected, in this experiment, 94.65% of reads mapped to protein-coding genes.

**Table 1:** Number of reads sequenced and mapped to the maize genome.

Sample	mf-S1	mf-S2	mf-S3	mf-F1	mf-F2	mf-F3
Raw reads	48,863,676	41,618,406	52,747,946	42,662,400	37,764,564	52,672,038
Clean reads	46,846,113	39,818,034	50,133,992	40,932,843	36,226,365	50,256,789
Clean ratio(%)	95.87	95.67	95.04	95.95	95.93	95.41
Q20(%)	94.77	94.47	95.05	94.78	94.81	94.76
GC-content(%)	56	55	55	55	55	57
Mapped unique reads	33,875,783	29,862,200	36,813,652	30,461,754	27,724,415	36,231,191
Mapped multi reads	1,170,976	946,392	1,296,876	1,337,016	970,614	1,187,026
Mapping ratio(%)	77.17	80.00	79.47	80.15	81.81	77.39

Noted:mf-F: Male inflorescence of fertility, mf-S: Male inflorescence of sterility

### 2.3. Identification of the DEGs in sterile and fertile samples

Genes with similar expression patterns may also share similar functions, such as being involved in the same metabolic processes or pathways in the developmental stage[24]. To identify the function of genes, researchers use similar expression patterns as an analytical strategy to cluster genes, thereby providing clues as to the function of unknown genes[25].

DEG analysis showed that most of the detected genes were exclusively expressed through down-regulation in the male sterile line. The detected DEGs were characterized using biased information, calculated as the differences between male sterile and male fertility in flowers by using the log expression ratios (trimmed mean of M values).

**Fig 3:**
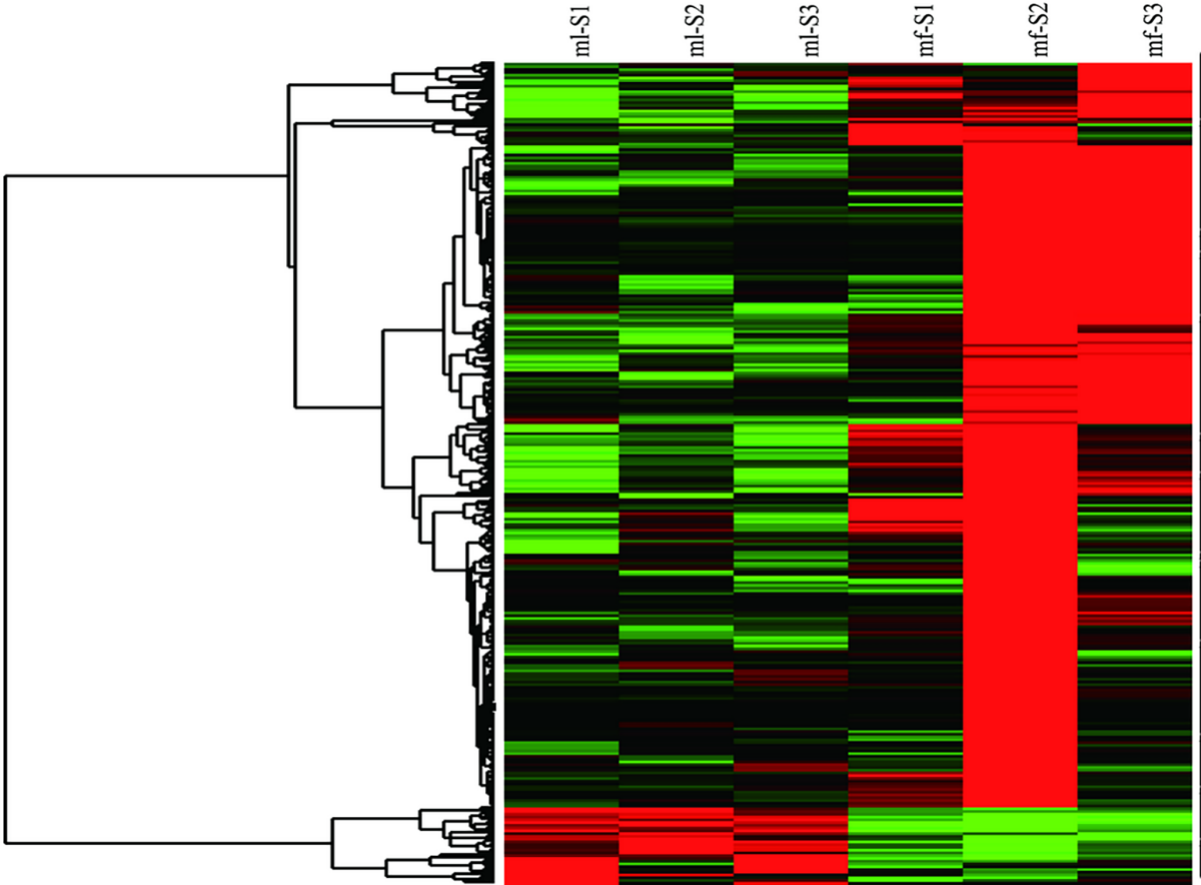
Hierarchical cluster analysis of gene expression based on the log ratio fold-change data. Noted:mf-F: Male inflorescence of fertility, mf-S: Male inflorescence of sterility.

To gain insights into the differential gene expression patterns between the sterile and fertile *712C-ms22* mutants in maize, we performed hierarchical clustering based on gene expression patterns to identify clusters with functional enrichment (Fig 3). DEGs were characterized as those genes with an RPKM value greater than or equal to 1 in at least one of the genotypes and with a false discovery rate (FDR)-adjusted P-value of less than 0.05. Using this significance level,we identified 395 DEGs between the sterile and fertile plants (log2FC>5 and q-value<0.05)(S1 Table). We found that gene expression levels in fertile plants showed only small differences, whereas large differences were observed in the gene expression levels in sterile plants. Gene expression levels were calculated using the RPKM values. The DEGs between sample groups were defined using the fold-change values of the normalized RPKM expression values.

Most of these genes were expressed as down-regulated in the male sterile line. The relationship of all the significant DEGs was visualized in a heat map as shown in Fig 3.When comparing the sterile and fertile mutant plants of *712C*-*ms22*,384 DEGs (log2FC>5 and P-value<0.05) were detected, including nine up-regulated genes and 375 down-regulated genes. The DEGs common to both genotypes were mainly involved in signal transduction, metabolism, transport and transcriptional regulation. Most of the DEGs were unique for each comparison group. Both gene ontology (GO) and Kyoto Encyclopedia of Genes and Genomes (KEGG) analyses were conducted for these DEGs. The functional classification of DEGs was also carried out(S1 Table, Fig 3).

### 2.4. Functional classification of genes using GO and KEGG analyses

**Fig 4:**
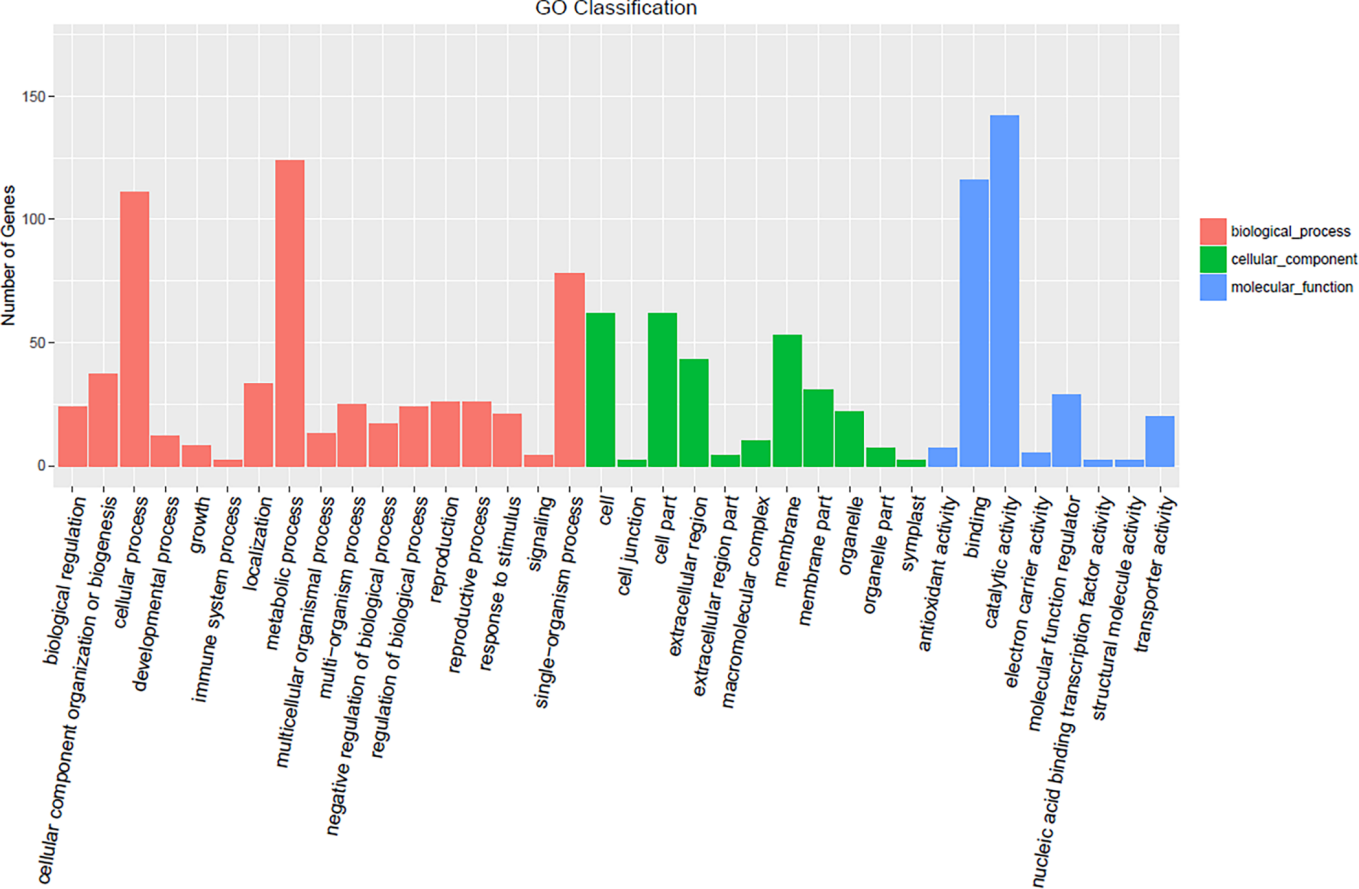
Gene ontology (GO) classifications of differentially expressed genes (DEGs) in the fertile and sterile plants.

GO is an internationally standardized gene function classification system used to describe the properties of genes and their products in any organism. This classification system contains three ontologies:biological process, cellular component and molecular function[26].The application of the GO assignment system to DEGs can assist in understanding the distribution of gene functions at a macro level[27–28]. In this study, plant GO slim annotation was conducted using Blast2GO software (http://www.blast2go.com/b2ghome). The 1206 DEGs (P-value<0.05)were enriched in 37 functional terms.The five most highly enriched GO terms were found to be catalytic activity(GO:0043086), metabolic process(GO:0008152), cellular process(GO:0009987), binding(GO:0005488) and single-organism process(GO:0044699).All unigenes were then assigned to the three main GO categories (biological process, cellular component and molecular function)(S2 Table, Fig 4).

Based on sequence homology, the 1206 DEGs were annotated into 36 functional categories, including 17 biological processes, 11 cellular components and eight molecular functions(Fig 4). Among the cellular component categories, “cell part and membrane” was the main functional group. Among the molecular function categories, “catalytic activity” was the main functional group, followed by “molecular function regulator” and “carbohydrate binding”.

A GO term was considered significantly enriched if the corrected P-value was below 0.05. In total, 242 DEGs(P-value<0.05) were annotated by gene ontology to belong to 19 functional categories. These genes were similarly classified into 19 categories by clusters of orthologous groups of proteins annotation. Fig 4 shows the data from the GO analysis of the regulated genes in the 712C-ms22plants(S1 and S2 Tables, Fig 4).

Other significantly enriched GO terms were identified when comparing the sterile and fertile plants. Some DEGs were involved in carbohydrate metabolism, and these may be transcription factors that are able to regulate processes such as pollen development, the elimination of reactive oxygen species, cellular signal transduction and programmed cell death in plants.These included “pollen tube growth” (GO: 0009860), “pollen tube development” (GO: 0048868),“sexual reproduction” (GO:0019953),“cell differentiation” (GO: 0030154), “cell tip growth”(GO: 0009932),“developmental growth” (GO: 0048589), “peroxidase activity” (GO: 0004601) and“antioxidant activity”(GO: 0016209) in the male inflorescence mutants of 712C-ms22(S4 Table).

The DEGs common to both genotypes were mainly involved in signal transduction, metabolism, transport and transcriptional regulation. Most of the DEGs were unique for each comparison group. Both GO and KEGG analyses were conducted for these DEGs. The functional classification of DEGs was also carried out. KEGG pathway analysis provides valuable classifications for studying the complex biological functions of genes.

**Fig 5:**
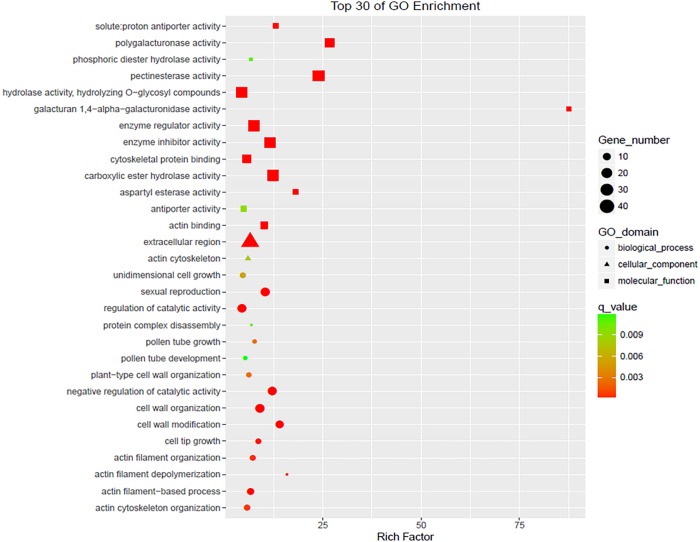
Top 30 of GO enrichment.

In this study, KEGG analysis revealed that pentose and glucoronate interconversions(zma00040)were the most significantly enriched DEGs in the male inflorescence 712C-ms22 mutant.Other significantly enriched KEGG pathways involved the following five terms: “nitrogen metabolism”(zma00910),“phenylpropanoid biosynthesis”(zma00940), “plant–pathogen interactions”(zma04626),“starch and sucrose metabolism”(zma00500) and“oxidative phosphorylation”(zma00190) (Table 2,S3 Table, Fig 5,.).

**Table 2:** Significantly enriched pathway terms identified by RNA-seq analysis.

Term	Simple number	Background number	Corrected P-value	Rich factor
Pentose and glucoronate interconversions	4	30	4.50E-06	23.57
Nitrogen metabolism	2	28	0.0016	12.62
Phenylpropanoid biosynthesis	3	91	0.0061	5.82
Plant–pathogen interaction	2	66	0.0174	5.35
Starch and sucrose metabolism	3	101	0.0087	5.25
Oxidative phosphorylation	2	88	0.037	4.01

The scatter plot of the differential gene KEGG enrichment analysis was shown as follows. Only the top 30 KEGG pathways in the top concentration were shown in the map.

### 2.5.Analysis of DEGs potentially related to male sterility

Carbohydrate and energy level metabolism is an important pathway during the process of plant growth, asit provides the required energy and carbon sources for the plant’s main physiological functions[29]. Defects in metabolic pathways may be a cause of male sterility in plants[30–33]. In this study, many DEGs were found to be involved in carbohydrate and energy metabolism as well as hydrolase activity and enzyme regulator activity. For example, 28 DEGs participated in carbohydrate and energy metabolism,26 in enzyme regulator activity and 35 in hydrolase activityin *712C*-*ms22* when comparing sterile and fertile plants.

**Fig 6:**
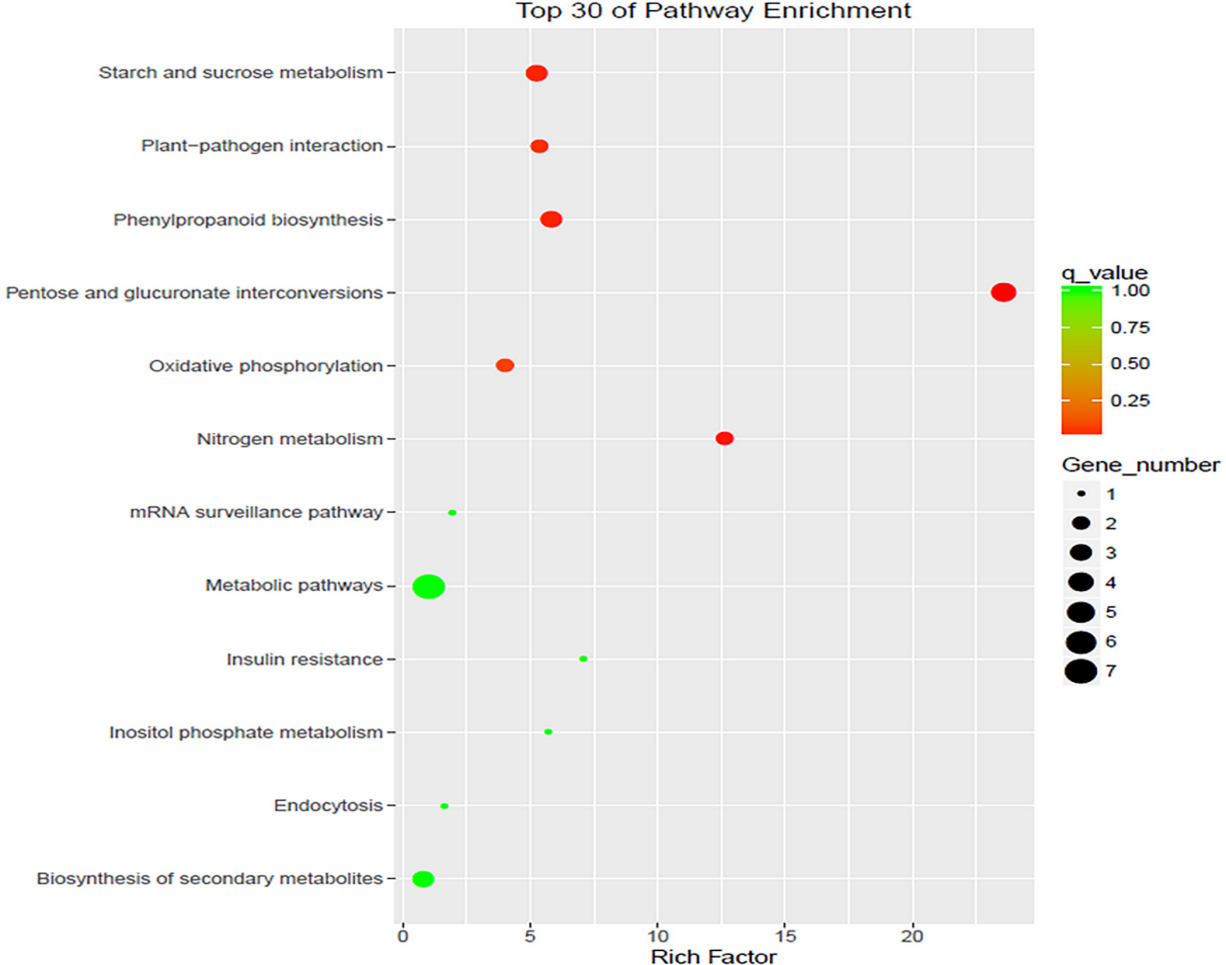
Top 30 pathways identified bypathway enrichment.

To further investigate these results, line plots of the top 30 genes were drawnand confirmed that thesegenes had similar patterns that were down-regulatedin expression in the male sterile line(S4 Table,Fig 5, Fig 6).

Transcription factors are essential for the regulation of gene expression. Changes in gene transcription are associated with changes in the expression of transcription factors. Our results showed that 17 DEGs participated in the process of sexual reproduction(GO: 0019953), sevenin cell tip growth(GO: 0009932), 10 in pollen tube growth and development(GO: 0009860), sevenin cell growth(GO: 0016049)and eightin cell differentiation(GO: 0030154),all of which were down-regulated when comparing the sterile and fertile plants.Moreover, 10 DEGs were found to be involved in the regulation of pollen development, while 30 DEGs participated in cell wall modification and organization and 15 DEGs participated in cell growth and differentiation, all of which were down-regulated when comparing the sterile and fertile *712C*-*ms22* plants(S1 Table, S3 Table).

We also found other DEGs potentially related to male sterility in *712C*-*ms22*. In total, 21 DEGs were related to the elimination of reactive oxygen species, among which 18 were down-regulated and three were up-regulated in *712C*-*ms22* when comparing the sterile and fertile plants. In addition, 10 DEGs associated with actin binding were detected, and 12 DEGs associated with calcium ion binding were found to be down-regulated(Table 3).

**Table 3:** DEGs potentially related to male sterility.

Gene ID	Up/Down	Gene annotation	log2FC
GRMZM2G380650	up	pollen development,developmental	8.973945
GRMZM2G020701	up	cell periphery,cell wall protein	8.493977
GRMZM2G103214	up	Aquaporin NIP1,transporter activity	8.01352
GRMZM2G160683	up	enzyme regulator activity	7.707771
AC196978.5	up	hydrolase activity,metabolic process	7.933834
GRMZM2G067096	up	antioxidant activity	7.75105
GRMZM2G364349	down	catalytic activity,pectate lyase	-12.7888
GRMZM2G312827	down	Calmodulin-binding protein MPCBP	-8.63515
GRMZM2G005643	down	Pollen allergen Phl,	-11.8225
GRMZM2G007708	down	sexual reproduction	-12.1751
GRMZM2G347047	down	Pollen allergen Phl, sexual reproduction	-9.30817
GRMZM2G089699	down	CDPK protein, Expansin-B11(EXPB11),reproduction	-11.0907

### 2.6.Analysis of DEGs by Qrt-PCR

**Fig 7:**
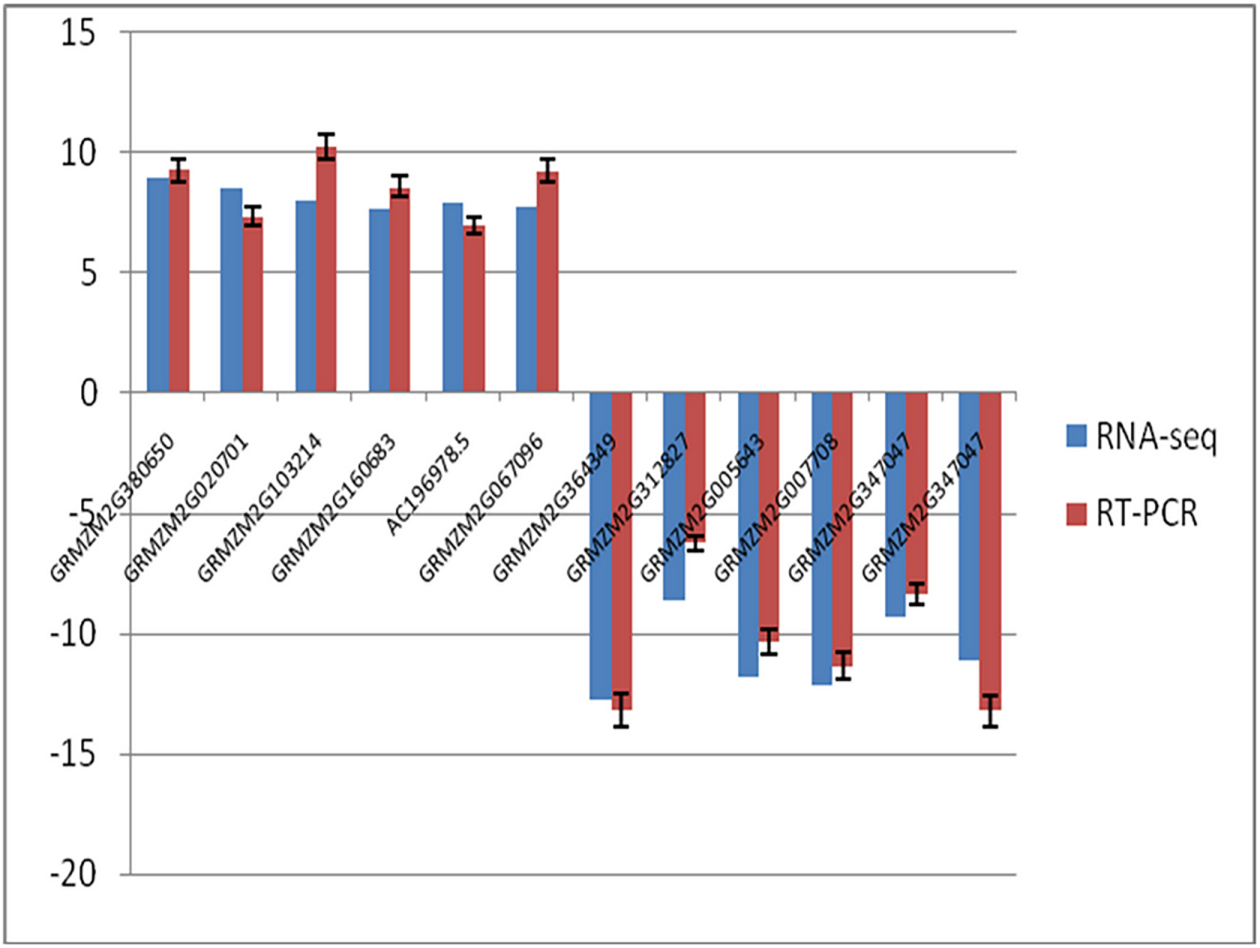
Qrt-PCR validation of 12randomly selected differentially expressed genes (DEGs) detected by RNA-seq.

According to the results of our functional and metabolic pathway analysis combined with the previously reported literature, 12 DEGs were randomly selected for Qrt-PCR analysis using the same sample as that used for RNA-seq. The expression patterns of the 12 DEGs were found to be consistent between Qrt-PCR and RNA-seq (Table 3;Fig 7), with a coincidence rate of 87.51% between the datasets, confirming that the RNA-seq results were reliable.

## 3.Discussion

GMS is common among most plants, and GMS plant material plays an important role in the production of hybrid crop seeds. To increase the yield as well as to overcome hybridization barriers, studies of maize gamete development, the pollen tube journey and fertilization mechanisms were initiated more than a century ago[33–35].

Previous studies have used transcriptional profiling or proteomic analysis to detect DEGs from sterile and fertile male lines[36–39]. During the process of plant growth and development, aberrant gene expression in any of the developmental stages relating to fertility (i.e., stamen development, male gametophyte development, pollen formation, pollen nutrient support and pollen tube elongation) can induce male sterility[40–41]. In the present study, RNA-seq was performed, and the transcripts of the staminate inflorescences between sterile and fertile males in *ms22* were compared using Illumina sequencing technology. We identified 395 DEGs between the sterile and fertile plants (log2FC>5 and P-value<0.05), among which 386 DEGs were down-regulated and 9 DEGs were up-regulated in *712C-ms22*(S1). To validate the RNA-seq data, qRT-PCR was performed on 12 of these genes and consistent results were obtained on both platforms.

The spatial and temporal regulation of the expression of genes relating to programmed cell death metabolic pathways, which mediate tapetum degeneration and anther dehiscence, has been proven in many studies[42–44]. In our analysis, we found that cell wall loosening was a prominent feature in *ms22*,involving pectatelyase, polygalacturonase, expansins, cellulose and glucan endo-1 3-beta-glucosidase5. Polygalacturonaseis known to be involved in anther dehiscence, pollen mother cell wall degradation and pollen tube growth[45]. Pectatelyase plays a role in pollen wall development and pollen tube growth[46].

In this study, fasciclin-like arabinogalactan protein 7(AGP7),GPI-anchored protein,COBRA-like protein 4, pentatricopeptide repeat superfamily protein and cytochrome coxidase polypeptide were annotated among the DEGs[47]. The callose wall is temporarily synthesized by callose synthase 5 (Cal5) that requires microsporecallose deposition to prevent cell fusion, and consequently, a Cal5 knockout mutant shows reduced fertility. Callose(beta-1,3-glucan) is a polysaccharide that separates developing pollen grains, preventing their walls from fusing[48–50]. The callose wall is broken down in a timely manner torelease microspores into the locules. Thus, the mistiming of callose degradation leads to malesterility.Beta-1,3-glucanase (callase), a programmed cell death-related gene expressed in the reproductive organs, is secreted from tapetum cells and causes callose degradation[51].

In this study, arabinogalactan protein (AGP)-encoding genes were detected among the DEGs that play an important role in the development of pollen, such as GPI-anchored proteins, GPI transamidase, COBRA-like proteins encoding GPI-anchored proteins and fasciclin-like *AGP6*,*AGP7*,*AGP16*,*AGP20* and *AGP17*.AGPsparticipate in cell expansion, division, seed germination and pollen tube growth and guidance[52–55]. Calcium gradient-mediated pollen tube growth is one of the best-characterized metabolic systems[56]. Several studies have demonstrated that Ca2+, a messenger in cellular signal transduction, functions asapivotal regulator of the cell life cycle,including cell division, differentiation and apoptosis[57]. The germination of the male gametophyte (the pollen grain) and proper elongation of the growing tube are essential processes in the sexual reproduction of plants, which involves many signaling pathways that guide male sperm cells to their target, the haploid egg cell (the female gametophyte)[58]. In this study,the expression of genes relating to calcium gradient-mediated pollen tube growth was detected in *ms22*, such as calcium-dependent protein kinase 2, calcium-binding protein 39, calcium-dependent protein kinase ZmCPK11, calcium-binding EF-hand protein, putative calcium-dependent lipid-binding (CaLB domain) family protein and calcium ion-binding protein. The calcium-binding proteins act as calcium sensors and relay calcium signals[59]. Major calcium sensors have been reported, such as calcium-dependent protein kinase, CaM, CBL proteins, CDPK protein and CML protein.Pollen development depended on multiple signaling pathways, in which calmodulin was a key element. In *Arabidopsis*, CIPK19 is expressed specifically in pollen grains and controls pollen polarity[60–61].

The MADS-box transcription factor controls the specification of stamen primordia.MADS-box transcription factor 1 was detected by our transcriptome analysis. Zinc-finger family proteins play an important role in flower development[62]. The *C2H2* zinc-finger family protein and *Cys2/His2* zinc-finger transcription factor were found to be significantly activated during anther development in plants[63].

The final developmental stage of pollen maturation includes a programmed desiccation process for enhancing pollen-geminating efficiency. In this study, genes related to this process were detected, including pollen-specific proteins (*C13*, *SF3)*, late embryogenesis abundant proteins (*EMB564*, *D-34*, *Lea14-A*) and dehydrin proteins (*DHN1*, *DHN13*). Additionally, the NADP reductase, ribonuclease, flap endonuclease, exosome complex exonuclease and sucrose cleavage protein were all detected by our transcriptome analysis. The study of the molecular mechanisms mediating male sterility in maize by comprehensive and systematic transcriptome profiling will provide valuable insights into the biological and developmental processes that occur throughout the life cycle of a plant66. We report DEGs that might be associated with the male sterility of *ms22* in maize, but their specific functions require further studies.

Male sterility has been widely studied in response to both its biological significance and commercial use in hybrid seed production. Genic male sterility occurs when lesions in nuclear-encoded genes disrupt normal male gametogenesis. Anthers develop through a series of highly coordinated steps that are well-defined at the morphological level[64].

Recent research in maizehas revealed that some mutants with anther identity defects,such as *ms-si-355* and *ems71990*, lack anthers within spikelets at the time when immature anthers normally exist. Furthermore, the mutants *ms25*, *ms26* and *ems-63131* exhibit highly irregular anther differentiation.It was found that microsporangia and all cell layers typical of the wild-type anther wall fail to differentiate.The *ms8*, *ms23*, *ms25*, *ms26*, *ms32* and *msca1* mutants each showed abnormalities in anther wall development. Mutant *ms8* showed meiocyte and tapetum degradation, *ms32*showed anther wall layer defects and undifferentiated cell layers, and *msca1-ms6064*showed anther identity defects[65]

MS23 regulates the specification and development of the tapetum in maize. Encoding an anther-specific predicted basic helix-loop-helix (bHLH) transcription factor required for tapetal differentiation.Microarray transcript profiling demonstrates a more severe developmental disruption in *ms23-ref* than in *ms23* anthers, which possess a different bHLH defect[66].

In the *ms8* mutant, tapetal cells become vacuolated and subsequently degrade. Interestingly, the *ms8* mutant also exhibits several mild defects, including an excess number of epidermal cells that are shorter than normal, fewer tapetal cells that are larger than normal, and excess callose accumulation during meiosis[67].

In this study, 39 DEGs relating to actin cytoskeleton organization were expressed in *ms22* including *actin-depolymerizing factor 1* (*ADF1*), *actin-depolymerizing factor 2* (*ADF2*) and *actin-depolymerizing factor 3* (*ADF3*). ADF regulates the actin cytoskeleton in *Arabidopsis* and is involved in polarized tip growth[68]. As an actin-binding protein, it affects the cytoskeleton architecture dynamics and plays a role in the regulation of F-actin filament assembly[69].

The development of the male reproductive organ includes sporophytic cell division through mitosis and male gametogenesis through meiosis. Dehydrins or late embryogenesis abundant proteins protect the pollen from desiccation. Mitotic and meiotic-specific gene destruction during the cell division process can lead to male sterility[70–71]. In this study, we identified 17 DEGs relating to reproduction in the *ms22*mutant and 25 DEGs relating to sexual reproduction in this mutant,including cell division cycle proteins(*cdt2*, *cdt48*, *cdt50*),elongation factor Ts and cell division protein AAA of the ATPase family. Interestingly, the expression of this DEG was down-regulated in the male sterile maize in our study.

## 4.Conclusions

Using RNA-seq technology, we performed global transcriptome sequencing analysis of the *ms22* mutant of male inflorescenceto compare sterility and fertility at the V10 developmental stage in maize. Our results demonstrated that additive gene expression patterns were prevalent in the staminate inflorescence development stages. This complementary effect may optimize gene expression levels and may play a fundamental role in the male inflorescence of maize.

Our analysis revealed that the male sterility of the *ms22*mutant in maize may be related to the regulated expression of some key DEGs, such as those genes involved in carbohydrate and energy metabolism, transcription factors, ribonuclease, pollen-specific kinase, reactive oxygen species elimination, cellular signal transduction, programmed cell death and reproductive development regulation. Our findings offer new insights into the molecular mechanisms of male sterility in maize. Future studies will focus on cloning and on determining the transgenic function of candidate genes associated with maize GMS.

This analysis revealed some essential genes responsible for pollen development and pollen tube elongation. Our findings provide useful reference data for male sterility in maize and new insights into the global mechanisms responsible for this phenotype.

## Materials and methods

### Preparation of plant materials

The *712C*-*ms22*mutant strain (also known as 7712C-ms22 and U140G) was provided by the Maize Genetics Cooperation Stock Center. Three biological replicates of each of the *ms22* mutant materials, *712C*-*ms22*andB73, were planted at the Jiangpu Experimental Station, Nanjing Agricultural University, Nanjing, Jiangsu, China. At developmental stage V10, the male ear was relatively tender, and the anther in the glume could be detected by microscopic examination of the pollen grains, which could be distinguished between the sterile and fertile male ears. Young sterile and fertile floral material was clipped from *712C*-*ms22*plants and then immediately frozen in liquid nitrogen and stored at ˗80°C prior to use in RNA-seq and subsequent experiments.

### Total RNA extraction and Illumina deep sequencing

Total RNA was extracted using RNAiso Plus Total RNA extraction reagent (TaKaRa, Dalian, China) following the manufacturer’s instructions, and the RIN number was measured as an indicator of RNA integrity using an Agilent Bioanalyzer 2100 (Agilent Technologies, Santa Clara, CA, USA). Qualified total RNA was further purified by anRNAClean XP Kit (Cat A63987, Beckman Coulter,Inc.Kraemer Boulevard, Brea, CA,USA)and an RNase-Free DNase Set (Qiagen, GmBH, Hilden, Germany). RNA samples with an RNA integrity number≥8, as assigned by the BioAnalyzer, were used for microarray analysis[27]. After purification, total RNA was sequenced and then subjected to mRNA isolation, frag mentation, first strand and second strand cDNA synthesis, terminal repair, 3'terminal plus A, connection joint and enrichment.Finally, a complete sequence sample library was constructed.

### Data analysis of RNA-seq

In this study,TopHat (version2.0.9) was applied for splice mapping of the pre-processed reads to the genome.Two mismatches were allowed, and each read was allowed multiple hits≤2,based on the length of the gene and the number of RPKM calculated for each gene[15]. For the three biological replicates, the uniquely mapped reads in each gene were counted using HTSeqversion5.4 software and the genes with RPKM values consistently >0 or 0.25 were used for further analysis. The mapped reads were then assembled with Cufflinks (http://cufflinks.cbcb.umd.edu/) [8–9].

### Identification of DEGs in *ms22*

The number of fragments for each gene was counted by Stringtie (version1.3.0),after comparison with TopHat and were then normalized by the TMM (trimmed mean of the M values) method. Edge R was applied to analyze gene differences between samples. P-value multiple hypothesis tests were corrected to control for the p-value threshold by controlling the false discovery rate (FDR). To identify the DEGs, the DESeq R package (1.10.1) was employed using a model based on a negative binomial distribution with default settings[27]. The multiplier of differential expression was calculated according to the FPKM value.The genes with RPKM values consistently >0 or 0.25 were included for further analysis. An FDR value≤0.05 and a log2-fold-change≥1 were used as the thresholds to judge the significance of gene expression differences[12].

### GO annotation, COG annotation and KEGG enrichment pathway analysis

A hypergeometric test was applied to select GO entries that were significantly enriched in DEGs compared with the whole genome background. Using the GO database, genes can be classified according to the biological processes they participate in, the components of the cells, and the function of the molecules. GO and functional enrichment(http://www.geneontology.org/) analyses were conducted on all of the DEGs using the Goatools software(https://github.com/tanghaibao/goatools), with a q-value ≤0.05 defining significant enrichment of a GO term in DEGs[26].

Metabolic pathway analysis was performed on all identified DEGs in the KEGG database(http://www.genome.jp/kegg/genes.html) using BlastX/BlastPand KOBAS (http://kobas.cbi.pku.edu.cn/home.do)[14–15] The functional classification of the clusters detected in the RNA-seq data of *712C*-*ms22* sterile and fertile young floral material was conducted on all identified DEGs using the Blastx software in the STRING9.0 database (http://string-db.org/) [24].

### qRT-PCR analysis

qRT-PCR analysis was used to verify the RNA-seq gene expression pattern. Reactions comprised 500 ng of total RNA, 1 μl of 10 mMdNTPs (Bioline, London, UK) and 1 μl of oligo-dT(20) primers (Invitrogen, Carlsbad, CA, USA), up to a final volume of 11 μl by the addition of RNase-free H_2_O. Reactions were performed at 65°C for 5 min, followed by 50°C for 60 min and were then inactivated at 70°C for 15 min.

The qRT-PCR primers were designed based on reference unigene sequences with the Primer Premier5.0 software. The Fast Sybr^R^Green Master Mix (Applied Biosystems,Foster City, CA, USA) was employed, according to the manufacturer’s instructions, in a reaction volume of 10 μl. Real-time PCR was conducted on an ABI 7500/7500 Fast Real-Time PCR system(Applied Biosystems).PCR conditions included initial denaturation for 2 min at 95°C, followed by 40 cycles of denaturation at 95°C for 30 s,hybridizationat 60°Cfor 40 s, and elongation at 68°C for 10 s. The *actin2* gene was used as an internal control. The 2^-ΔΔct^ method was used to calculate the relative level of gene expression, and the B73 sample served as a control. A relative level of gene expression greater than 1 was considered to indicate up-regulation, and less than 1 indicateddown-regulation. All qRT-PCR reactions were performed with the three biological replicates.

## Supporting information

S1 Table Number of differentially expressed genes between sterility and fertility. (XLS)

S2 Table Gene Ontology functional annontation of differentially expressed genes between sterility and fertility. (XLS)

S3 Table GO enrichment expressed genes (DEGs) between sterility and fertility. (XLS)

S4 Table Top 30 pathways of GO enrichment between sterility and fertility. (XLS)

## References

[1] Ranum P, PenaRosas JP, GarciaCasal MN.Global maize production, utilization, and consumption. Ann.N.Y.Acad.Sci. 2014;1312(1):105–12. 24650320 10.1111/nyas.12396

[2] Gong Fangping, Yang Le, Tai Fuju, Hu Xiuli, Wang Wei. “Omics” of Maize Stress Response for Sustainable Food Production: Opportunities and Challenges. OMICS A Journal of Integrative Biology. 2014;18(12):714–32. 10.1089/omi.2014.0125 25401749 PMC4253144

[3] Wei Fusheng, Zhang Jianwei, Zhou Shiguo, He Ruifeng, Schaeffer Mary, Wing Rod A. The Physical and Genetic Framework of the Maize B73 Genome. Plos Genetics.2009;5(11):e1000715 10.1371/journal.pgen.1000715 19936061 PMC2774505

[4] Muraya MM, Schmutzer T, Ulpinnis C, Scholz U.Targeted Sequencing Reveals Large-Scale Sequence Polymorphism in Maize Candidate Genes for Biomass Production and Composition. PLoS One.2015;10(7):e0132120 10.1371/journal.pone.0132120 26151830 PMC4495061

[5] Brady SM, Provart NJ.Web-Queryable Large-Scale Data Sets for HypothesisGeneration in Plant Biology.The Plant Cell.2009;21:1034–51. 10.1105/tpc.109.066050 19401381 PMC2685637

[6] Schnable PS, Springer NM. Progress toward understanding heterosis in crop plants. Annu Rev Plant Biol. 2013;64:71–8. 10.1146/annurev-arplant-042110-103827 23394499

[7] Rhee SunJu, Seo Minseok, Jang YoonJeong, Cho Seoae, gPyoLee Gun.Transcriptome profiling of differentiallyexpressed genes in floral buds and flowersof male sterile and fertile lines inwatermelon. BMC Genomics.2015;16:914 10.1186/s12864-015-2186-9 26552448 PMC4640349

[8] Kim D, Pertea G, Trapnell C, Pimentel H, Kelley R, Salzberg SL. TopHat2: accurate alignment of transcriptomes in the presence of insertions,deletions and gene fusions. Genome Biol. 2013;14 (4):R36 10.1186/gb-2013-14-4-r36 23618408 PMC4053844

[9] Trapnell C, Roberts A, Goff L, Pertea G, Kim D, Kelley DR, et al Differential gene and trans -cript expression analysis of RNA-seq experiments with TopHat and Cufflinks. Nat Protoc. 2014;7: 562–78. 10.1038/nprot.2012.016 PMC3334321 22383036

[10] Guo SG, Zheng Y, Joung JG, Liu SQ, Zhang ZH, Crasta OR, et al Transcriptome sequencing and comparative analysis of cucumber flowers with different sex types. BMC Genomics.2010,; 11:569–78. 10.1186/1471-2164-11-569 20565788 PMC2897810

[11] Zhou H, Zhou M, Yang Y, Li J, Gu L, Zhou L, etal *RNase ZS1* processes *UbL40* mRNAs and controls thermosensitive genic male sterility in rice.Nat.Commun. 2014,;5, 4884–97. 10.1038/ncomms5884 25208476

[12] Scott C, Rajandeep S, Candice N, Hirsch C, Natalia L, Shawn M, etal An Expanded Maize Gene Expression Atlas based on RNA Sequencing and its Use to Explore Root Development. Plant Genome,2015;9(1):679–92. 10.3835/plantgenome2015.04.0025 27898762

[13] Wang Z, Gerstein M, Snyder M. RNA-Seq: A revolutionary tool for transcriptomics.Nat. Rev. Genet. 2009;10, 57–63. 10.1038/nrg2484 19015660 PMC2949280

[14] Trapnell C, Roberts A, Goff L, Pertea G, Kim D, Kelley DR, et al Differential gene and trans -cript expression analysis of RNA-seq experiments with TopHat and Cufflinks. Nat Protoc. 2012;7: 562–78. 10.1038/nprot.2012.016 22383036 PMC3334321

[15] Trapnell C, Williams BA, Pertea G, Mortazavi A, Kwan G, van Baren MJ, et al Transcript assembly and quantification by RNA-Seq reveals unannotated transcripts and isoform switching during cell differentiation. Nat Biotechnol. 2010;28: 511–15. 10.1038/nbt.1621 20436464 PMC3146043

[16] Wang LG, Wang SQ, Li W. RSeQC: quality control of RNA-seq experiments. Bioinformatics. 2012;28:2184–85. 10.1093/bioinformatics/bts356 22743226

[17] Yan XH, Dong CH, Yu JY, Liu WH, Jiang CH, Liu J, et al Transcriptome profile analysis of young floral buds of fertile and sterile plants from the self-pollinated offspring of the hybrid between novel restorer line NR1 and Nsa CMS line in Brassica napus. BMC Genomics. 2012;14: 226–38. 10.1186/1471-2164-14-26 PMC3556089 23324545

[18] Liu C, Ma N, Wang PY, Fu N, Shen HL. Transcriptome sequencing and De Novo analysis of a cytoplasmic male sterile line and its near-isogenic restorer line in chili pepper (*Capsicum annuum L*.). PLoS One.2013;8(6):e65209 10.1371/journal.pone.0065209 23750245 PMC3672106

[19] Wei Mingming, Song Meizhen, Fan Shuli, Yu Shuxun.Transcriptomic analysis of differentially expressed genes during anther development in genetic male sterile and wild type cotton bydigital gene-expression profiling. BMC Genomics.2013,14:97–06. 10.1186/1471-2164-14-97 23402279 PMC3599889

[20] Min Haowei, Chen Chengxuan, Wei Shaowei, Shang Xiaoling, Chen Huabang, Xie Qi, et al Identification of Drought Tolerant Mechanisms in Maize Seedlings Based on Transcriptome Analysis of Recombination Inbred Lines.Front Plant Sci.2016;1080:1–19. 10.3389/fpls.2016.01080 PMC4961006 27507977

[21] Anwar A, Mehdi M. Role of genetically engineered system of male sterility in hybrid production of vegetables. J Phytology. 2009;1(6):448–60.

[22] Vedel F, Pla M, Vitart V, Gutierres S, Chetrit P, Depaepe R. Molecular-basis of nuclear and cytoplasmic male-sterility in higher-plants. Plant Physiol Bioch.1994,32(5):601–18.

[23] Chen ZJ. Genomic and epigenetic insights into the molecular bases of heterosis. Nat Rev Genet. 2013;14:471–82. 10.1038/nrg3503 23752794

[24] Mao X, Cai T, Olyarchuk JG, Wei L. Automated genome annotation and pathway identific -ation using the KEGG Orthology (KO) as a controlled vocabulary. *Bioinformatics*.2005,;21, 3787–93. 10.1093/bioinformatics/bti430 15817693

[25] Rajandeep S, Sekhon RB, Candice N.,Hirsch CL, Natalia L, Shawn M, et al Maize gene atlas developed by RNA sequencing and comparative evaluation of transcriptomes based on RNA sequencing and microarrays. PLoS ONE. 2013;8:E61005 10.1371/journal.pone.0061005 23637782 PMC3634062

[26] Young MD, Wakefield MJ, Smyth GK, Oshlack A. Gene ontology analysis for RNA- seq: Accounting for selection bias. *Genome Biol*. 2010,;11, R14 10.1186/gb-2010-11-2-r14 20132535 PMC2872874

[27] Anders S, Pyl PT, Huber W. HTSeq–A Python framework to work with highthroughput sequencing data. Bioinformatics. 2014;31(2):166–79. 10.1093/bioinformatics/btu638 25260700 PMC4287950

[28] Anders S, Huber W. Differential expression analysis for sequence count data. Genome Biol.2010,;11, R106 10.1186/gb-2010-11-10-r106 20979621 PMC3218662

[29] Zhou L, Lan W, Chen B, Fang W, Luan S. A calcium sensor-regulated protein kinase, Calic-Neurin B-likeprotein-interavting protein kinase19, is required for pollen tube growth and polarity. Plant Physiol.2015;167(4):1351–60. 10.1104/pp.114.256065 25713341 PMC4378171

[30] Xie CT, Wei DM, Tian HQ. Advances in cell biological researches on male sterility of higher plants. J Plant Physiol Mol Biol. 2006;32(1):17–23. 16477126

[31] Anwar A, Mehdi M. Role of genetically engineered system of male sterility in hybrid production of vegetables. J Phytology. 2009;1(6):448–60.

[32] Sheoran IS, Sawhney VK. Proteome analysis of the normal and Ogura (ogu) CMS anthers of Brassica napus to identify proteins associated with male sterility. Botany. 2010;88: 217–230.

[33] Suzuki H, RodriguezUribe L, Xu JN, Zhang JF. Transcriptome analysis of cytoplasmic male sterility and restoration in CMS-D8 cotton. Plant Cell Rep.2013;32(10):1531–42. 10.1007/s00299-013-1465-7 23743655

[34] Gomes S, Civetta A.Hybrid male sterility and genome-wide misexpression of male reproductive proteases.Scientific Reports.,2015;5:11976 10.1038/srep11976 26146165 PMC4491705

[35] Tang HB, Wang XY, Bowers JE, Ming R, Alam M, Paterson AH. Unraveling ancient hexap -loidy through multiply-aligned angiosperm gene maps. Genome Res. 2008;18:1944–54. 10.1101/gr.080978.108 18832442 PMC2593578

[36] Varnier AL, Mazeyrat-Gourbeyre F, Sangwan RS, Clement C. Programmed cell death progre -ssively models the development of anther sporophytic tissues from the tapetum and is triggered in pollen grains during maturation. J Struct Biol. 2005;152(2):118–28. 10.1016/j.jsb.2005.07.011 16256370

[37] Wang CS, Hsu SW, Hsu YF. new insights into desiccation-associated gene regulation by Lilium longiflorum ASR during pollen maturation and in transgenic Arabidopsis. Int Rev Cel Mol Bio. 2013;301:37–64. 10.1016/B978-0-12-407704-1.00002-6 23317817

[38] Wang DX, Oses-Prieto JA, Li KH, Fernandes JF, Burlingame AL, Walbot V. The male sterile 8 mutation of maize disrupts the temporal progression of the transcriptome and results in the mis-regulation of metabolic functions. Plant J. 2010;63(6):939–51. 10.1111/j.1365-313X.2010.04294.x 20626649 PMC2974755

[39] Xu C, Liu Z, Zhang L, Zhao C, Yuan S, Zhang F. Organization of actin cytoskeleton during meiosis I in a wheat thermo-sensitive genic male sterile line. Protoplasma. 2013;250(1):415–22. 10.1007/s00709-012-0386-6 22350736

[40] Bosch M, Cheung AY, Hepler PK. Pectin methylesterase, a regulator of pollen tube growth. Plant Physiol. 2005;138(3):1334–46. 10.1104/pp.105.059865 15951488 PMC1176407

[41] Zheng BB, Wu XM, Ge XX, Deng XX, Grosser JW, Guo WW. Comparative transcript profil -ing of a male sterile cybridpummelo and its fertile type revealed altered gene expression related to flower development. PLoS One.2012;7(8):e43758 10.1371/journal.pone.0043758 22952758 PMC3429507

[42] Wu J, Wang S, Gu Y, Zhang S, Publicover SJ, Franklin VE.Self-incompatibility in Papaver rhoeas activates nonspecific cation conductance permeable to Ca2+ and K+, Plant Physiol. 2011;155:963–73. 10.1104/pp.110.161927 21177472 PMC3032480

[43] Wilson ZA, Zhang DB. From Arabidopsis to rice: Pathways in pollen development. J Exp Bot. 2009;60(5):1479–92. 10.1093/jxb/erp095 19321648

[44] Jiang JJ, Yao LN, Yu YJ, Liang Y, Jiang JX, Ye NH. PECTATE LYASE-LIKE 9from Brassica campestris is associated with intine formation. Plant Sci.2014;229:66–75. 10.1016/j.plantsci.2014.08.008 25443834

[45] Sander L, Child R, Ulvskov P, Albrechtsen M, Borkhardt B. Analysis of a dehiscence zone endo- polygalacturonase in oilseed rape (Brassica napus) and Arabidopsis thaliana: evidence for roles in cell separation in dehiscence and abscission zones, and in stylar tissues during pollen tube growth. Plant Mol Biol. 2001;46(4):469–79. 11485203 10.1023/a:1010619002833

[46] Gorguet B, Schipper D, van Lammeren A, Visser RGF, van Heusden AW. *ps-2*,the gene responsi -ble for functional sterility in tomato, due to non-dehiscent anthers, is the result of a mutation in a novel polygalacturonasegene.Theor Appl Genet. 2009;118(6):1199–209. 10.1007/s00122-009-0974-9 19219598

[47] Grotewold E, Chamberlin M, Snook M, Siame B, Butler L, Swenson J. Engineering secondary metabolism in maize cells by ectopic expression of transcription factors. Plant Cell. 1998;10: 721–40. 9596632 PMC144024

[48] Enns LC, Kanaoka MM, Torii KU, Comai L, Okada K, Cleland RE. Two callose synthases, GSL1 and GSL5, play an essential and redundant role in plant and pollen development and in fertility. Plant Mol Biol.2005;58(3):333–49. 10.1007/s11103-005-4526-7 16021399

[49] Dong XY, Hong ZL, Sivaramakrishnan M, Mahfouz M, Verma DPS. Callose synthase (CalS5) is required for exine formation during microgametogenesis and for pollen viability in Arabidopsis. Plant J. 2005;42(3):315–28. 10.1111/j.1365-313X.2005.02379.x 15842618

[50] Chen XY, Liu L, Lee E, Han X, Rim Y, Chu H, et al The Arabidopsis Callose Synthase Gene GSL8 Is Required for Cytokinesis and Cell Patterning. Plant Physiol. 2009;150(1):105–13. 10.1104/pp.108.133918 19286936 PMC2675722

[51] Miura K, Lee J, Gong Q, Ma S, Jin J.B, Yoo CY.Miura T, Hasegawa PM. *SIZ1* regulation of phosphate starvation-induced root architecture remodeling involves the control of auxin accumula -tion. *Plant Physiol*. 2011,;155, 1000–12. 10.1104/pp.110.165191 21156857 PMC3032448

[52] Bosch M, Cheung AY, Hepler PK. Pectin methylesterase, a regulator of pollen tube growth. Plant Physiol. 2005;138(3):1334–46. 10.1104/pp.105.059865 15951488 PMC1176407

[53] Fu Y, Wu G, Yang ZB. Rop GTPase-dependent dynamics of tip-localized F-actin controls tip growth in pollen tubes. J Cell Biol. 2001;152(5):1019–32. 11238457 10.1083/jcb.152.5.1019 PMC2198818

[54] Chen CB, Marcus A, Li WX, Hu Y, Calzada JPV, Grossniklaus U, et al The Arabidopsis ATK1 gene is required for spindle morphogenesis in male meiosis. Development. 2002;129 (10): 2401–09. 11973272 10.1242/dev.129.10.2401

[55] Bou Daher F, van Oostende C, Geitmann A. Spatial and temporal expression of actin depoly -merizing factors ADF7 and ADF10 during male gametophyte development in Arabidopsis thaliana. Plant Cell Physiol. 2011;52(7):1177–92. 10.1093/pcp/pcr068 21632657

[56] Messerli MA, Creton R, Jaffe LF, Robinson KR.Periodic increases in elongation rate precede increases in cytosolic Ca2+ during pollen tube growth.Dev. Biol.2000;222:84–98. 10.1006/dbio.2000.9709 10885748

[57] Steinhorst L, Kudla J. Calcium—a central regulator of pollen germination and tube growth. Bba-Mol Cell Res. 2013;1833(7):1573–81. 10.1016/j.bbamcr.2012.10.009 23072967

[58] Taylor LP, Hepler PK. Pollen germination and tube growth, Annu. Rev. Plant Physiol. Plant Mol. Biol. 1997;48:461–91. 10.1146/annurev.arplant.48.1.461 15012271

[59] Carafoli E. Calcium signaling: a tale for all seasons. Proc Natl Acad Sci USA. 2002;99: 1115–22. 10.1073/pnas.032427999 11830654 PMC122154

[60] Wu J, Wang S, Gu Y, Zhang S, Publicover SJ, Franklin VE.Self-incompatibility in Papaver rhoeas activates nonspecific cation conductance permeable to Ca2+ and K+, Plant Physiol. 2011;155:963–73. 10.1104/pp.110.161927 21177472 PMC3032480

[61] Steinhorst L, Kudla J. Calcium—a central regulator of pollen germination and tube growth. Bba-Mol Cell Res. 2013;1833(7):1573–81. 10.1016/j.bbamcr.2012.10.009 23072967

[62] Kobayashi A, Sakamoto A, Kubo K, Rybka Z, Kanno Y, Takatsuji H. Seven zinc-finger trans -cription factors are expressed sequentially during the development of anthers in petunia. Plant J. 1998;13(4):571–76. 9680999 10.1046/j.1365-313x.1998.00043.x

[63] Jan A, Maruyama K, Todaka D, Kidokoro S, Kidokoro S, Yoshimura E, et al OsTZF1, a CCCH-tandem zinc finger protein, confers delayed senescence and stress tolerance in rice by regulating stress-related genes. Plant Physiol.2013,;161, 1202–16. 10.1104/pp.112.205385 23296688 PMC3585590

[64] Skibbe DS, Schnabl PS.MALE STERILITY IN MAIZE. Maydica.2005;50: 367–76.

[65] judmi, Timofejeva, Davi, Cande WilliamZacheus.Cytological Characterization and AllelismTestingof Anther Developmental Mutants Identified in a Screen of Maize Male Sterile Lines.G3 Genesgenetics.2013;3(2):231. 10.1534/g3.112.004465 PMC3564984 23390600

[66] Nan GuoLing, Zhai Jixian, Arikit Siwaret, Morrow Darren, Nguyen Nhi, Walbot Virginia, et al MS23, a master basic helix-loop-helix factor, regulates the specification and development of tapetum in maize. Development.2017;144(1):163 10.1242/dev.140673 27913638

[67] Wang DX, OsesPrieto JA,KHLi,Fernandes JF, Burlingame AL.male sterility 8 mutation of maize disrupts the temporal progression of the transcriptome and results in mis-regulation of metabolic functions. Plant J.2010;63:939–51. 10.1111/j.1365-313X.2010.04294.x 20626649 PMC2974755

[68] Maciver SK, Hussey PJ.The ADF/cofilin family: actin-remodeling proteins.Genome Biol,.2002,;3(5):653–65. 10.1186/gb-2002-3-5-reviews3007 PMC139363 12049672

[69] Bou Daher F, van Oostende C, Geitmann A. Spatial and temporal expression of actin depoly -meri-zing factors ADF7 and ADF10 during male gametophyte development in Arabidopsis thaliana. Plant Cell Physiol. 2011;52(7):1177–92. 10.1093/pcp/pcr068 21632657

[70] Charfeddine S, Saidi MN, Charfeddine M, GargouriBouzid R.Genome-wide identification and expression profiling of the late embryogenesis abundant genes in potato with emphasis on dehyd -rins.Molecular Biology Reports.2015;42(7):1163–75. 10.1007/s11033-015-3853-2 25638043

[71] Li X, Cao J.Late Embryogenesis Abundant (LEA) Gene Family in Maize: Identification, Evolu -tion, and Expression Profiles.Plant Molecular Biology Reporter.2016;34(1):1–14.

